# Crystal structure of 1-mesityl-3-methyl-4-phenyl-1*H*-1,2,3-triazol-3-ium iodide

**DOI:** 10.1107/S2056989015023403

**Published:** 2015-12-12

**Authors:** Daniel Canseco-González, Juventino J. García, Marcos Flores-Alamo

**Affiliations:** a Facultad de Química, Universidad Nacional Autónoma de México, Circuito Interior, Ciudad Universitaria, Mexico City, 04510, Mexico

**Keywords:** crystal structure, triazolium salt, mesityl group, C—H⋯I hydrogen bonds

## Abstract

In the cation of the title salt, C_18_H_20_N_3_^+^·I^−^, the mesityl and phenyl rings are inclined to the central triazolium ring by 61.39 (16) and 30.99 (16)°, respectively, and to one another by 37.75 (15)°. In the crystal, mol­ecules are linked *via* C—H⋯I hydrogen bonds, forming slabs parallel to the *ab* plane. Within the slabs there are weak π–π inter­actions present involving the mesityl and phenyl rings [inter-centroid distances are 3.8663 (18) and 3.8141 (18) Å].

## Related literature   

For classical Arduengo-type imidazol-2-yl­idene *N*-heterocyclic carbenes (NHCs), see: Arduengo *et al.* (1995 ▸); Mathew *et al.* (2008 ▸). For similar 1-mesityl-3-methyl-4-phenyl-1*H*-1,2,3-triazol-3-ium structures and some complexes, see: Saravanakumar *et al.* (2011 ▸); Hohloch *et al.* (2011 ▸, 2013 ▸); Shaik *et al.* (2013 ▸).
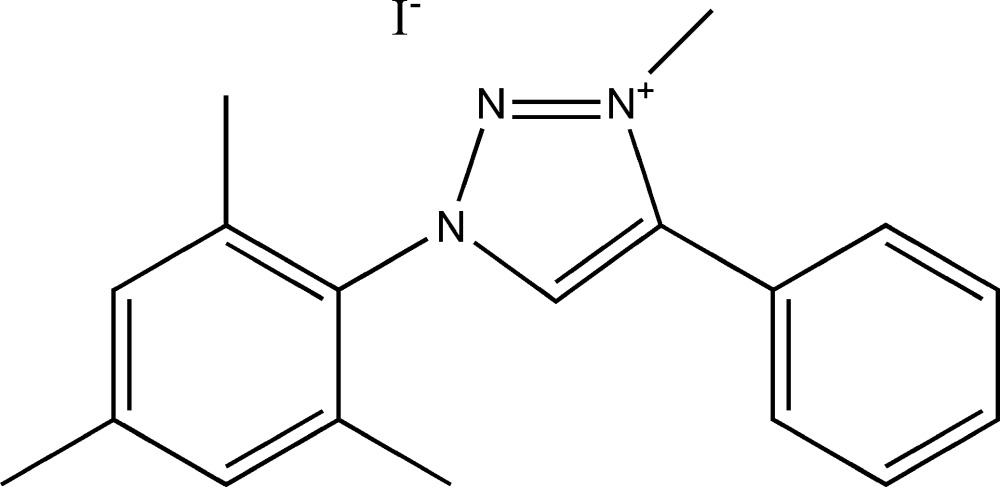


## Experimental   

### Crystal data  

C_18_H_20_N_3_^+^·I^−^
*M* *_r_* = 405.27
Monoclinic, 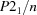

*a* = 7.6704 (3) Å
*b* = 9.9341 (3) Å
*c* = 22.8541 (10) Å
β = 98.982 (4)°
*V* = 1720.09 (12) Å^3^
*Z* = 4
Mo *K*α radiation
μ = 1.86 mm^−1^
*T* = 130 K
0.14 × 0.08 × 0.02 mm

### Data collection  

Agilent Xcalibur Atlas Gemini diffractometer
Absorption correction: analytical (*CrysAlis RED*; Agilent, 2013 ▸) *T*_min_ = 0.864, *T*_max_ = 0.963
8948 measured reflections
4073 independent reflections
3374 reflections with *I* > 2σ(*I*)
*R*_int_ = 0.031

### Refinement  

*R*[*F* ^2^ > 2σ(*F* ^2^)] = 0.034
*wR*(*F* ^2^) = 0.084
*S* = 1.16
4073 reflections
203 parameters
H-atom parameters constrained
Δρ_max_ = 1.05 e Å^−3^
Δρ_min_ = −0.57 e Å^−3^

### 

Data collection: (*CrysAlis PRO*; Agilent, 2013 ▸); cell refinement: (*CrysAlis RED*; Agilent, 2013 ▸); data reduction: (*CrysAlis RED*; program(s) used to solve structure: *SHELXS2014* (Sheldrick, 2008 ▸); program(s) used to refine structure: *SHELXL2014* (Sheldrick, 2015 ▸); molecular graphics: *Mercury* (Macrae *et al.*, 2008 ▸); software used to prepare material for publication: *WinGX* (Farrugia, 2012 ▸).

## Supplementary Material

Crystal structure: contains datablock(s) global, I. DOI: 10.1107/S2056989015023403/su5254sup1.cif

Structure factors: contains datablock(s) I. DOI: 10.1107/S2056989015023403/su5254Isup2.hkl

Supporting information file. DOI: 10.1107/S2056989015023403/su5254Isup3.cml

**Fig. 1.**
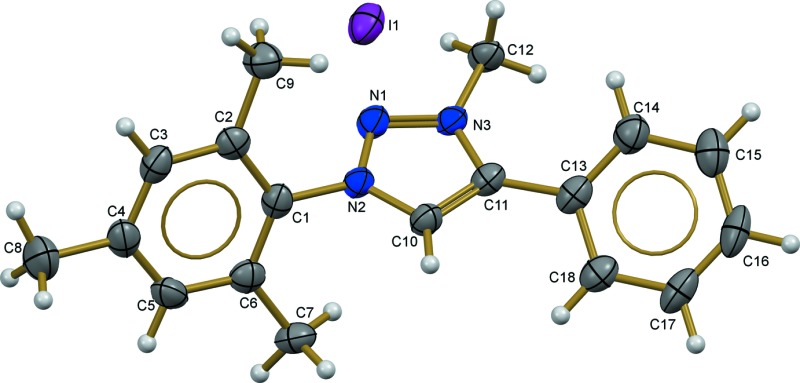
The molecular structure of the title salt, with atom labelling. Displacement ellipsoids are drawn at the 50% probability level.

**Fig. 2.**
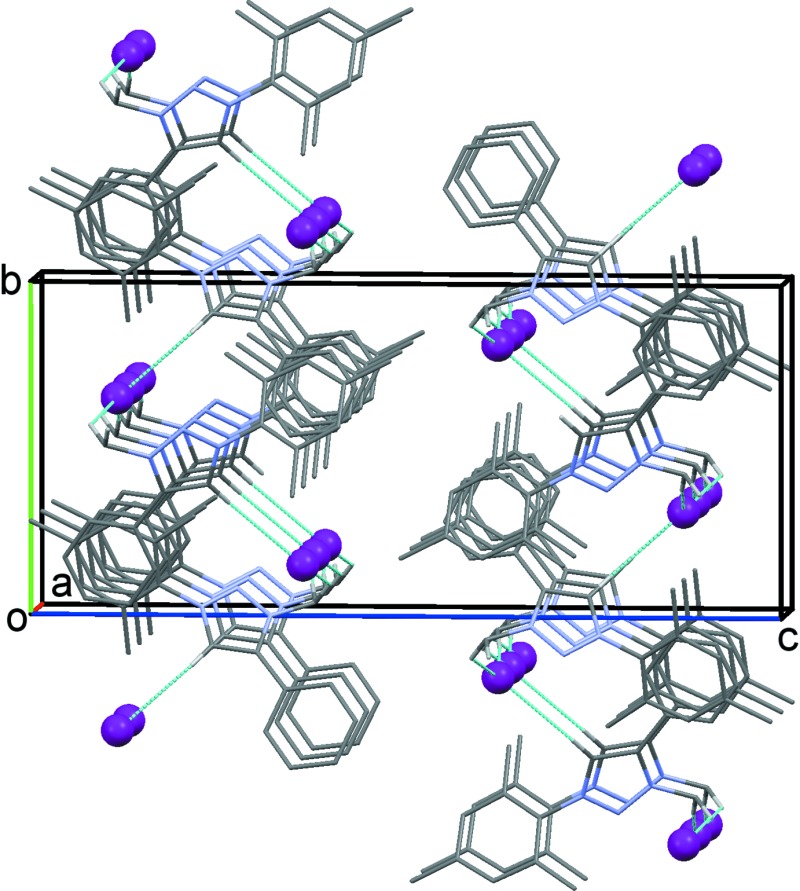
A view along the a axis of the crystal packing of the title compound. The C—H···I hydrogen bonds are shown as dashed lines (see Table 1). H atoms not involved in these interactions have been omitted for clarity.

. DOI: 10.1107/S2056989015023403/su5254fig1.tif
The mol­ecular structure of the title salt, with atom labelling. Displacement ellipsoids are drawn at the 50% probability level.

a . DOI: 10.1107/S2056989015023403/su5254fig2.tif
A view along the *a* axis of the crystal packing of the title compound. The C—H⋯I hydrogen bonds are shown as dashed lines (see Table 1). H atoms not involved in these inter­actions have been omitted for clarity.

CCDC reference: 1440705

Additional supporting information:  crystallographic information; 3D view; checkCIF report

## Figures and Tables

**Table 1:** Hydrogen-bond geometry (Å, °)

*D*—H⋯*A*	*D*—H	H⋯*A*	*D*⋯*A*	*D*—H⋯*A*
C10—H10⋯I1^i^	0.95	3.12	4.049 (3)	168
C12—H12*A*⋯I1^ii^	0.98	3.20	3.916 (3)	131
C12—H12*B*⋯I1	0.98	3.22	4.172 (3)	163

Symmetry codes: (i) 
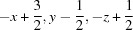
; (ii) 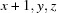.

## supplementary crystallographic information

### S1. Commentary

Mesoionic 1,2,3-triazol-5-ylidenes bear only one nitro­gen adjacent to the carbene bonding site and are more basic than the classical Arduengo-type imidazol-2-yl­idene NHCs (Arduengo *et al.*, 1995; Mathew *et al.*, 2008). This type of triazolyl­idene has recently been applied for the development of a variety of organometallic complexes, specially directed towards catalytic purposes (Saravanakumar *et al.*, 2011; Hohloch *et al.*, 2011,2013); Shaik *et al.*, 2013).

In the cation of the title salt, Fig. 1, the central triazolium ring (N1—N3/C10/C11) is inclined to the mesityl (C1—C6) and phenyl (C13—C18) rings by 61.39 (16) and 30.99 (16) °, respectively, while the two six-membered aromatic rings are inclined to one another by 37.75 (15) °.

In the crystal, molecules are linked *via* C—H···I hydrogen bonds forming slabs parallel to the *ab* plane (Table 1 and Fig. 2). Within the slabs there are slipped parallel π-π inter­actions present involving the mesityl and phenyl rings: *Cg*2···*Cg*3^i^ = 3.8663 (18) Å [where *Cg*2 and *Cg*3 are the centroids of rings C1—C6 and C13—C18, inter­planar distance = 3.6798 (13) Å, slippage = 1.595 Å; symmetry ocde: (i) − *x* + 3/2, *y* + 1/2, − *z* + 1/3], and *Cg*2···*Cg*3^ii^ = 3.8141 (18) Å [inter­planar distance = 3.5739 (13) Å, slippage = 1.797 Å; symmetry ocde: (ii) − *x* + 5/2, *y* + 1/2, −*z* + 1/2]; see Fig. 2.

### S2. Synthesis and crystallization

Synthesis of 1-mesityl-4-phenyl-1,2,3-triazole

2-azido-1,3,5-tri­methyl­benzene (868 mg, 5.4 mmol), and phenyl­acetyl­ene (1000 mg, 4.9 mmol) were suspended in a mixture of water (16.0 ml) and ^*t*^BuOH (16.0 ml). To the previous mixture CuSO_4_ (10.6 mg, 0.05 mmol), and sodium ascorbate (97 mg, 0.50 mmol) were added and stirred for 24 h at 100 °C. The reaction mixture was allowed to cool and ^*t*^BuOH was evaporated off. The resulted mixture was extracted with CH_2_Cl_2_ (2 × 100 ml). The combined organic phases were washed with water (2 × 60 ml), brine (2 × 50 ml), dried over MgSO_4_ and evaporated to dryness. The residue was washed with pentane (50 ml) to afford the crude triazole as an off brown solid. The crude product was recrystallized from hot acetone to give the corresponding pure triazole (yield: 1100 mg, 85%). ^1^H NMR (CDCl_3_, 300 MHz): δ 7.93 (d, ^3^*J*_HH_ = 7.8 Hz, 2H, H_ar_), 7.84 (s, 1H, H_trz_), 7.47 (t, ^3^*J*_HH_ = 7.8 Hz, 2H, H_ar_), 7.36 (t, ^3^*J*_HH_ = 7.5 Hz, 1H, H_ar_), 7.01 (s, 2H, H_mes_), 2.37 (s, 3H, ArCH_3_), 2.02 (s, 6H, ArCH_3_). ^13^C{^1^H} NMR (CDCl_3_, 75 MHz): δ 147.5 (C_trz–Mes_), 140.0, 135.1, 133.5, 130.4, 129.1 (5 × C_ar_), 128.9 (C_trz-H_), 130.4, 128.8, 128.2 (3 × C_ar_), 21.1 (Ar—CH_3_), 17.3 (Ar—CH_3_).

Synthesis of 1-mesityl-3-methyl-4-phenyl-1*H*-1,2,3-triazol-3-ium iodide

A solution of 1-mesityl-4-phenyl-1,2,3-triazole (500 mg, 1.3 mmol) in MeCN (12 ml) was added CH_3_I (1.7 g, 12 mmol) and the mixture was stirred at 373 K for 48 h. The workup and purification were carried out according to the general method. The title compound was obtained as a white solid (yield: 612 mg, 80%). Colourless plate-like crystals were obtained by ???? - please complete.^1^H NMR (CDCl_3_, 300 MHz): δ 8.86 (s, 1H, H_trz_), 8.04 (m, 2H, Har), 7.58 (m, 3H, Har), 7.05 (s, 2H, Hmes), 4.58 (s, 3H, NCH3), 2.37 (s, 3H, ArCH3), 2.22 (s, 6H, ArCH3). ^13^C{^1^H} NMR (CDCl3, 75 MHz): δ 144.2 (Ctrz–Mes), 142.5, 134.4, 132.2, 130.4, 130.1, 129.9, 129.7, 121.2, (8 × Car), 40.7 (NCH3), 21.2 (Ar—CH3), 18.5 (Ar—CH3). Anal. Calcd. for C18H20IN3 *x* 1 H2O (423.28): C 51.44, H 5.24, N 9.93. Found: C 51.44, H 4.34, N 10.17.

### S3. Refinement details

Crystal data, data collection and structure refinement details are summarized in Table 2. The C-bound H atoms were placed in geometrically idealized positions and refined as riding on their parent atoms: C—H = 0.95–0.98 Å with *U*_iso_(H) = 1.5*U*_eq_(*C*-methyl) and 1.2*U*_eq_(C) for other H atoms.

### Crystal data

**Table:**

C_18_H_20_N_3_^+^·I^−^	*F*(000) = 808
*M**_r_* = 405.27	*D*_x_ = 1.565 Mg m^−^^3^
Monoclinic, *P*2_1_/*n*	Mo *K*α radiation, λ = 0.71073 Å
Hall symbol: -P 2yn	Cell parameters from 3748 reflections
*a* = 7.6704 (3) Å	θ = 4.5–29.3°
*b* = 9.9341 (3) Å	µ = 1.86 mm^−^^1^
*c* = 22.8541 (10) Å	*T* = 130 K
β = 98.982 (4)°	Plate, colourless
*V* = 1720.09 (12) Å^3^	0.14 × 0.08 × 0.02 mm
*Z* = 4	

### Data collection

**Table:**

Agilent Xcalibur Atlas Gemini diffractometer	4073 independent reflections
Graphite monochromator	3374 reflections with *I* > 2σ(*I*)
Detector resolution: 10.4685 pixels mm^-1^	*R*_int_ = 0.031
ω scans	θ_max_ = 29.3°, θ_min_ = 3.4°
Absorption correction: analytical (*CrysAlis RED*; Agilent, 2013)	*h* = −10→9
*T*_min_ = 0.864, *T*_max_ = 0.963	*k* = −13→12
8948 measured reflections	*l* = −20→30

### Refinement

**Table:**

Refinement on *F*^2^	0 restraints
Least-squares matrix: full	Hydrogen site location: inferred from neighbouring sites
*R*[*F*^2^ > 2σ(*F*^2^)] = 0.034	H-atom parameters constrained
*wR*(*F*^2^) = 0.084	*w* = 1/[σ^2^(*F*_o_^2^) + (0.0324*P*)^2^ + 0.4334*P*] where *P* = (*F*_o_^2^ + 2*F*_c_^2^)/3
*S* = 1.16	(Δ/σ)_max_ = 0.001
4073 reflections	Δρ_max_ = 1.05 e Å^−^^3^
203 parameters	Δρ_min_ = −0.57 e Å^−^^3^

### Special details

**Table:**

Geometry. All e.s.d.'s (except the e.s.d. in the dihedral angle between two l.s. planes) are estimated using the full covariance matrix. The cell e.s.d.'s are taken into account individually in the estimation of e.s.d.'s in distances, angles and torsion angles; correlations between e.s.d.'s in cell parameters are only used when they are defined by crystal symmetry. An approximate (isotropic) treatment of cell e.s.d.'s is used for estimating e.s.d.'s involving l.s. planes.

### Fractional atomic coordinates and isotropic or equivalent isotropic displacement parameters (Å^2^)

**Table:**

	*x*	*y*	*z*	*U*_iso_*/*U*_eq_	
C1	0.9473 (4)	0.6044 (3)	0.32285 (13)	0.0166 (6)	
C2	1.0174 (4)	0.7345 (3)	0.32716 (13)	0.0166 (6)	
C3	1.0440 (4)	0.7926 (3)	0.38314 (14)	0.0193 (7)	
H3	1.0952	0.8798	0.3878	0.023*	
C4	0.9986 (4)	0.7281 (3)	0.43240 (14)	0.0207 (7)	
C5	0.9244 (4)	0.6001 (3)	0.42536 (14)	0.0205 (7)	
H5	0.8905	0.5562	0.4587	0.025*	
C6	0.8984 (4)	0.5345 (3)	0.37081 (13)	0.0178 (6)	
C7	0.8235 (4)	0.3951 (3)	0.36552 (14)	0.0217 (7)	
H7A	0.7477	0.3814	0.3958	0.033*	
H7B	0.92	0.3293	0.3712	0.033*	
H7C	0.7538	0.3832	0.3261	0.033*	
C8	1.0311 (6)	0.7949 (4)	0.49185 (16)	0.0336 (9)	
H8A	1.1237	0.7462	0.5179	0.05*	
H8B	0.9222	0.7943	0.5093	0.05*	
H8C	1.0687	0.8881	0.4873	0.05*	
C9	1.0623 (4)	0.8130 (3)	0.27524 (15)	0.0224 (7)	
H9A	1.1435	0.8862	0.2896	0.034*	
H9B	0.9541	0.8507	0.2527	0.034*	
H9C	1.1186	0.7533	0.2496	0.034*	
C10	1.0035 (4)	0.4227 (3)	0.25127 (13)	0.0174 (6)	
H10	1.0827	0.3672	0.2768	0.021*	
C11	0.9446 (4)	0.4023 (3)	0.19207 (13)	0.0165 (6)	
C12	0.7388 (4)	0.5467 (3)	0.11771 (13)	0.0193 (7)	
H12A	0.8138	0.6043	0.0973	0.029*	
H12B	0.6313	0.5958	0.1228	0.029*	
H12C	0.707	0.4654	0.0942	0.029*	
C13	0.9856 (4)	0.2918 (3)	0.15337 (14)	0.0185 (7)	
C14	0.9941 (4)	0.3091 (3)	0.09329 (15)	0.0224 (7)	
H14	0.9724	0.3951	0.0755	0.027*	
C15	1.0344 (5)	0.2003 (3)	0.05942 (16)	0.0262 (8)	
H15	1.0361	0.2112	0.0182	0.031*	
C16	1.0722 (4)	0.0760 (3)	0.08598 (16)	0.0265 (8)	
H16	1.1002	0.0018	0.0629	0.032*	
C17	1.0694 (4)	0.0596 (3)	0.14553 (16)	0.0240 (7)	
H17	1.0984	−0.0253	0.1635	0.029*	
C18	1.0248 (4)	0.1657 (3)	0.17954 (15)	0.0207 (7)	
H18	1.0207	0.153	0.2205	0.025*	
I1	0.22531 (3)	0.67415 (2)	0.12660 (2)	0.02384 (9)	
N1	0.8211 (3)	0.5913 (3)	0.22025 (11)	0.0174 (5)	
N2	0.9259 (3)	0.5380 (2)	0.26597 (10)	0.0152 (5)	
N3	0.8348 (3)	0.5089 (2)	0.17589 (11)	0.0157 (5)	

### Atomic displacement parameters (Å^2^)

**Table:**

	*U*^11^	*U*^22^	*U*^33^	*U*^12^	*U*^13^	*U*^23^
C1	0.0163 (14)	0.0163 (15)	0.0157 (15)	0.0032 (13)	−0.0023 (11)	−0.0023 (13)
C2	0.0134 (14)	0.0184 (16)	0.0174 (15)	0.0047 (13)	0.0006 (11)	0.0015 (13)
C3	0.0193 (15)	0.0152 (15)	0.0223 (17)	0.0029 (13)	−0.0003 (12)	−0.0031 (13)
C4	0.0226 (16)	0.0223 (17)	0.0165 (16)	0.0075 (14)	0.0002 (12)	−0.0011 (14)
C5	0.0250 (16)	0.0217 (17)	0.0149 (15)	0.0047 (14)	0.0029 (12)	0.0048 (13)
C6	0.0167 (14)	0.0170 (15)	0.0189 (15)	0.0040 (13)	0.0005 (12)	0.0016 (13)
C7	0.0251 (16)	0.0163 (16)	0.0234 (17)	0.0000 (14)	0.0025 (13)	0.0055 (13)
C8	0.052 (2)	0.028 (2)	0.0201 (18)	0.0025 (18)	0.0015 (16)	−0.0050 (15)
C9	0.0242 (16)	0.0210 (17)	0.0214 (17)	−0.0020 (14)	0.0016 (13)	0.0014 (14)
C10	0.0198 (15)	0.0141 (15)	0.0177 (15)	0.0032 (13)	0.0010 (12)	0.0000 (12)
C11	0.0160 (14)	0.0130 (15)	0.0199 (16)	−0.0001 (12)	0.0014 (12)	−0.0003 (12)
C12	0.0232 (16)	0.0157 (16)	0.0163 (16)	0.0019 (13)	−0.0050 (12)	0.0008 (12)
C13	0.0156 (14)	0.0148 (15)	0.0242 (17)	0.0005 (13)	0.0006 (12)	−0.0021 (13)
C14	0.0246 (16)	0.0193 (17)	0.0225 (17)	0.0000 (14)	0.0013 (13)	−0.0035 (14)
C15	0.0267 (17)	0.0276 (19)	0.0244 (18)	−0.0022 (15)	0.0040 (14)	−0.0094 (15)
C16	0.0250 (17)	0.0199 (17)	0.035 (2)	−0.0024 (15)	0.0062 (14)	−0.0147 (15)
C17	0.0214 (16)	0.0141 (16)	0.036 (2)	0.0023 (14)	0.0021 (14)	−0.0059 (14)
C18	0.0194 (15)	0.0165 (16)	0.0255 (17)	−0.0009 (13)	0.0015 (13)	−0.0007 (14)
I1	0.02450 (13)	0.01763 (12)	0.03061 (14)	−0.00299 (9)	0.00815 (9)	−0.00440 (9)
N1	0.0191 (13)	0.0143 (13)	0.0179 (13)	0.0005 (11)	0.0004 (10)	−0.0018 (11)
N2	0.0171 (12)	0.0128 (12)	0.0143 (12)	0.0020 (11)	−0.0018 (10)	−0.0006 (10)
N3	0.0188 (12)	0.0112 (12)	0.0158 (12)	−0.0005 (11)	−0.0009 (10)	0.0000 (10)

### Geometric parameters (Å, º)

**Table:**

C1—C6	1.397 (4)	C10—C11	1.373 (4)
C1—C2	1.397 (4)	C10—H10	0.95
C1—N2	1.444 (4)	C11—N3	1.367 (4)
C2—C3	1.389 (4)	C11—C13	1.475 (4)
C2—C9	1.504 (4)	C12—N3	1.465 (4)
C3—C4	1.386 (5)	C12—H12A	0.98
C3—H3	0.95	C12—H12B	0.98
C4—C5	1.392 (5)	C12—H12C	0.98
C4—C8	1.498 (5)	C13—C14	1.395 (5)
C5—C6	1.393 (4)	C13—C18	1.400 (4)
C5—H5	0.95	C14—C15	1.392 (5)
C6—C7	1.497 (4)	C14—H14	0.95
C7—H7A	0.98	C15—C16	1.386 (5)
C7—H7B	0.98	C15—H15	0.95
C7—H7C	0.98	C16—C17	1.374 (5)
C8—H8A	0.98	C16—H16	0.95
C8—H8B	0.98	C17—C18	1.384 (4)
C8—H8C	0.98	C17—H17	0.95
C9—H9A	0.98	C18—H18	0.95
C9—H9B	0.98	N1—N3	1.320 (3)
C9—H9C	0.98	N1—N2	1.325 (3)
C10—N2	1.358 (4)		
			
C6—C1—C2	123.5 (3)	N2—C10—H10	126.9
C6—C1—N2	118.2 (3)	C11—C10—H10	126.9
C2—C1—N2	118.3 (3)	N3—C11—C10	104.3 (3)
C3—C2—C1	116.7 (3)	N3—C11—C13	126.5 (3)
C3—C2—C9	119.5 (3)	C10—C11—C13	129.2 (3)
C1—C2—C9	123.9 (3)	N3—C12—H12A	109.5
C4—C3—C2	122.4 (3)	N3—C12—H12B	109.5
C4—C3—H3	118.8	H12A—C12—H12B	109.5
C2—C3—H3	118.8	N3—C12—H12C	109.5
C3—C4—C5	118.6 (3)	H12A—C12—H12C	109.5
C3—C4—C8	120.3 (3)	H12B—C12—H12C	109.5
C5—C4—C8	121.1 (3)	C14—C13—C18	119.4 (3)
C4—C5—C6	122.0 (3)	C14—C13—C11	123.1 (3)
C4—C5—H5	119	C18—C13—C11	117.5 (3)
C6—C5—H5	119	C15—C14—C13	120.0 (3)
C5—C6—C1	116.8 (3)	C15—C14—H14	120
C5—C6—C7	120.3 (3)	C13—C14—H14	120
C1—C6—C7	122.9 (3)	C16—C15—C14	119.8 (3)
C6—C7—H7A	109.5	C16—C15—H15	120.1
C6—C7—H7B	109.5	C14—C15—H15	120.1
H7A—C7—H7B	109.5	C17—C16—C15	120.3 (3)
C6—C7—H7C	109.5	C17—C16—H16	119.8
H7A—C7—H7C	109.5	C15—C16—H16	119.8
H7B—C7—H7C	109.5	C16—C17—C18	120.6 (3)
C4—C8—H8A	109.5	C16—C17—H17	119.7
C4—C8—H8B	109.5	C18—C17—H17	119.7
H8A—C8—H8B	109.5	C17—C18—C13	119.8 (3)
C4—C8—H8C	109.5	C17—C18—H18	120.1
H8A—C8—H8C	109.5	C13—C18—H18	120.1
H8B—C8—H8C	109.5	N3—N1—N2	104.3 (2)
C2—C9—H9A	109.5	N1—N2—C10	112.2 (2)
C2—C9—H9B	109.5	N1—N2—C1	119.8 (2)
H9A—C9—H9B	109.5	C10—N2—C1	128.0 (2)
C2—C9—H9C	109.5	N1—N3—C11	113.1 (2)
H9A—C9—H9C	109.5	N1—N3—C12	116.7 (2)
H9B—C9—H9C	109.5	C11—N3—C12	130.2 (3)
N2—C10—C11	106.2 (3)		
			
C6—C1—C2—C3	2.3 (4)	C18—C13—C14—C15	2.5 (5)
N2—C1—C2—C3	−177.0 (3)	C11—C13—C14—C15	179.7 (3)
C6—C1—C2—C9	−177.0 (3)	C13—C14—C15—C16	−2.3 (5)
N2—C1—C2—C9	3.8 (4)	C14—C15—C16—C17	0.2 (5)
C1—C2—C3—C4	−2.1 (4)	C15—C16—C17—C18	1.6 (5)
C9—C2—C3—C4	177.2 (3)	C16—C17—C18—C13	−1.3 (5)
C2—C3—C4—C5	0.3 (5)	C14—C13—C18—C17	−0.7 (5)
C2—C3—C4—C8	179.6 (3)	C11—C13—C18—C17	−178.0 (3)
C3—C4—C5—C6	1.4 (5)	N3—N1—N2—C10	−0.7 (3)
C8—C4—C5—C6	−177.9 (3)	N3—N1—N2—C1	178.6 (2)
C4—C5—C6—C1	−1.2 (4)	C11—C10—N2—N1	0.5 (4)
C4—C5—C6—C7	178.0 (3)	C11—C10—N2—C1	−178.8 (3)
C2—C1—C6—C5	−0.7 (4)	C6—C1—N2—N1	119.6 (3)
N2—C1—C6—C5	178.6 (3)	C2—C1—N2—N1	−61.0 (4)
C2—C1—C6—C7	−180.0 (3)	C6—C1—N2—C10	−61.2 (4)
N2—C1—C6—C7	−0.7 (4)	C2—C1—N2—C10	118.1 (3)
N2—C10—C11—N3	0.0 (3)	N2—N1—N3—C11	0.7 (3)
N2—C10—C11—C13	−179.4 (3)	N2—N1—N3—C12	−176.8 (2)
N3—C11—C13—C14	33.1 (5)	C10—C11—N3—N1	−0.5 (3)
C10—C11—C13—C14	−147.6 (3)	C13—C11—N3—N1	179.0 (3)
N3—C11—C13—C18	−149.6 (3)	C10—C11—N3—C12	176.7 (3)
C10—C11—C13—C18	29.7 (5)	C13—C11—N3—C12	−3.9 (5)

### Hydrogen-bond geometry (Å, º)

**Table:**

*D*—H···*A*	*D*—H	H···*A*	*D*···*A*	*D*—H···*A*
C10—H10···I1^i^	0.95	3.12	4.049 (3)	168
C12—H12*A*···I1^ii^	0.98	3.20	3.916 (3)	131
C12—H12*B*···I1	0.98	3.22	4.172 (3)	163

Symmetry codes: (i) −*x*+3/2, *y*−1/2, −*z*+1/2; (ii) *x*+1, *y*, *z*.
