# Supplementary figures and images for: Crystal structure of 1-mesityl-3-methyl-4-phenyl-1H-1,2,3-triazol-3-ium iodide
Source: Acta Crystallogr E Crystallogr Commun. 2015 Dec 12;71(Pt 12):o1041–2. doi: 10.1107/S2056989015023403 (PMC4719969; doi:10.1107/S2056989015023403)

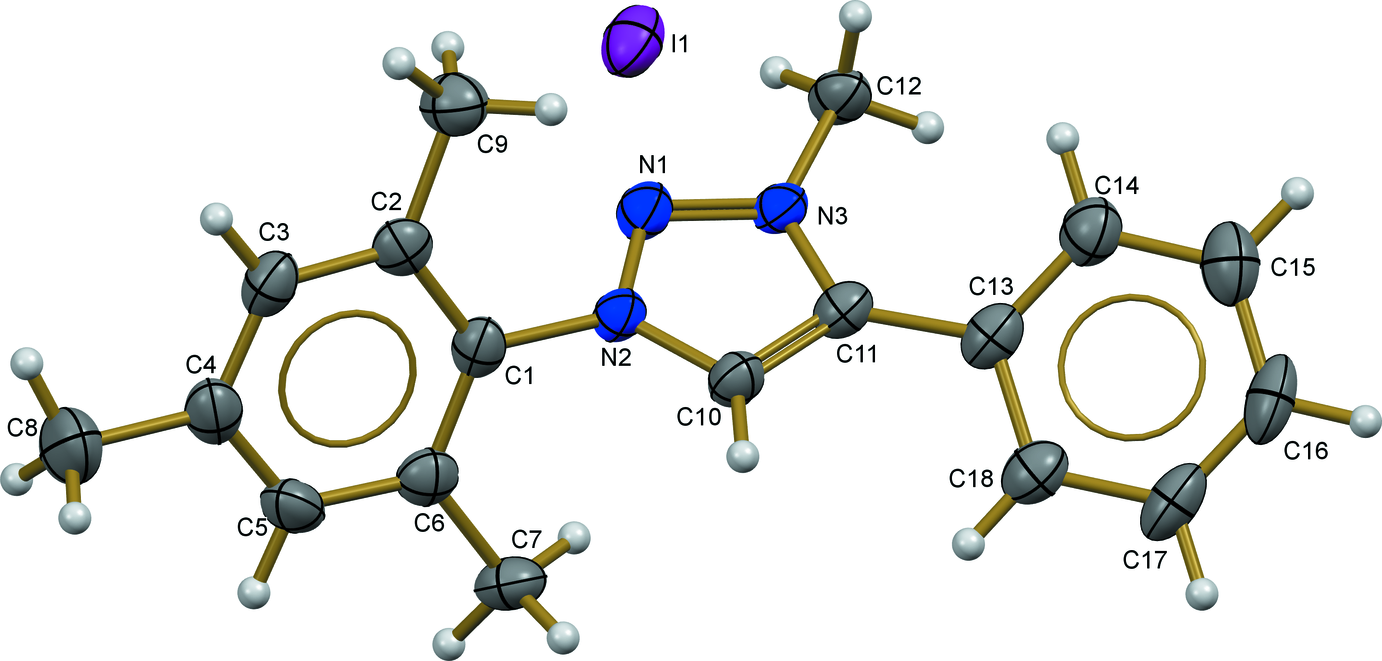

Supplement: Supplementary file 4 [file e-71-o1041-fig1.tif]

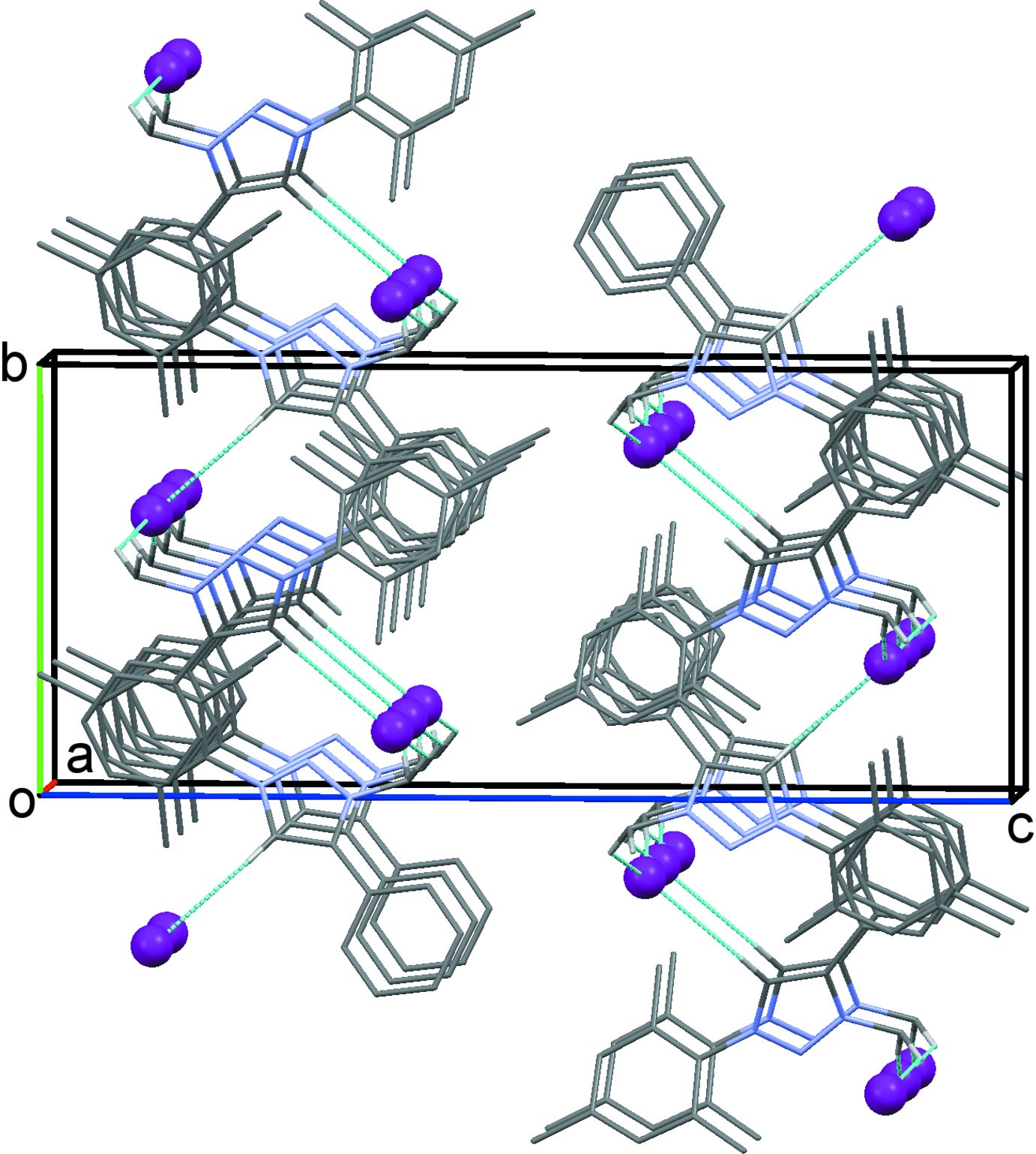

Supplement: Supplementary file 5 [file e-71-o1041-fig2.tif]
